# Characterization of non-linear mechanical behavior of the cornea

**DOI:** 10.1038/s41598-020-68391-7

**Published:** 2020-07-14

**Authors:** A. Ashofteh Yazdi, J. Melchor, J. Torres, I. Faris, A. Callejas, M. Gonzalez-Andrades, G. Rus

**Affiliations:** 1 Ultrasonics Lab, Department of Structural Mechanics, University of Granada, Politécnico de Fuentenueva, 18071 Granada, Spain; 2 Department of Biomedical Engineering, Islamic Azad University, Mashhad Branch, Mashhad, Iran; 3 Department of Statistics and Operations Research, University of Granada, Granada, Spain; 4 Instituto de Investigación Biosanitaria, Ibs.GRANADA, Granada, Spain; 5 Excellence Research Unit, “Modelling Nature” (MNat), University of Granada, Granada, Spain; 6 Maimonides Biomedical Research Institute of Cordoba (IMIBIC), Department of Ophthalmology, Reina Sofia University Hospital and University of Cordoba, Edificio IMIBIC, Av. Menéndez Pidal, s/n. 14004, Cordoba, Spain; 7 Massachusetts Eye and Ear and Schepens Eye Research Institute, Department of Ophthalmology, Harvard Medical School, Boston, MA USA

**Keywords:** Biomedical engineering, Diagnostic markers, Corneal diseases

## Abstract

The objective of this study was to evaluate which hyperelastic model could best describe the non-linear mechanical behavior of the cornea, in order to characterize the capability of the non-linear model parameters to discriminate structural changes in a damaged cornea. Porcine corneas were used, establishing two different groups: control (non-treated) and NaOH-treated (damaged) corneas (n = 8). NaOH causes a chemical burn to the corneal tissue, simulating a disease associated to structural damage of the stromal layer. Quasi-static uniaxial tensile tests were performed in nasal-temporal direction immediately after preparing corneal strips from the two groups. Three non-linear hyperelastic models (i.e. Hamilton-Zabolotskaya model, Ogden model and Mooney-Rivlin model) were fitted to the stress–strain curves obtained in the tensile tests and statistically compared. The corneas from the two groups showed a non-linear mechanical behavior that was best described by the Hamilton-Zabolotskaya model, obtaining the highest coefficient of determination (R^2^ > 0.95). Moreover, Hamilton-Zabolotskaya model showed the highest discriminative capability of the non-linear model parameter (Parameter A) for the tissue structural changes between the two sample groups (p = 0.0005). The present work determines the best hyperelastic model with the highest discriminative capability in description of the non-linear mechanical behavior of the cornea.

## Introduction

The cornea is the outermost layer of the eye, acting as a barrier against the external environment and as the main diopter of the visual system^1^. Diseases that affect the cornea are one of the main causes of blindness in the world, ranking among the three most prevalent worldwide^2^. Among those, corneal ectasia or corneal ectatic disorders stand as a main priority because of their incidence and impact in young population. These are corneal disorders, such as keratoconus or pellucid marginal corneal degeneration, that cause alterations in the corneal structure, leading to corneal topographical changes with decreased corneal thickness and abnormal corneal curvature^3^. This abnormal structure of the cornea, as a result of non-linear mechanical changes, finally causes visual impairment to the patient. Currently, the diagnose of corneal degenerations and other corneal diseases are mainly based on structural measurements of the cornea (i.e. curvature, thickness, etc.), while some studies covered in-vivo non-structural measurements using Oculus Corvis ST and Ocular Response Analyzer systems^4–6^. These new, non-invasive systems can analyze corneal biomechanical properties, such as corneal hysteresis, including an estimation of intraocular pressure. However, we propose that non-linear mechanical parameters can be obtained from non-invasive elastography or probing technologies such as non-linear torsional waves, non-linearity in probing, non-linearity by micro-indentation to be used in diagnostics in-vivo without removing the cornea^7–9^. Hence, quantifying the non-linear mechanical parameters of soft tissues like the cornea might become more specific diagnostic criteria than structural measurements. This is supported by the fact that non-linear mechanical characterization and non-linear model parameters are very sensitive realistic approaches to measure the tissue structural damages^7,8^. The feasibility of early diagnosis of corneal diseases such as keratoconus is however an open issue to be investigated in the future. This will depend on the precision of the nonlinearity parameter achievable by the selected probing technology, and thereof define the sensitivity and sensibility. Despite of that, non-linear elastic constants may be much more sensitive to specific diseases, i.e. expressing larger variations than linear ones, which might facilitate an early diagnosis^9^.

Thus, non-linear models might facilitate to understand how corneal diseases affect the structure and the mechanical behavior of the cornea, and how this leads to blurred vision, or even blindness, in combination with other strategies. In order to determine the direct effect on vision, these models should be combined with methods to calculate the changes in geometry and methods to trace the light through the optical system or wave-front calculations^10–12^. Moreover, non-linear models could improve the diagnosis of some corneal diseases such as keratoconus, in addition to facilitate the evaluation of specific therapeutic strategies like corneal collagen crosslinking^13^. To achieve this, measurements of the elastic or shear modulus in healthy and abnormal corneas are required. Many studies have measured the elastic modulus of healthy and keratoconus corneas using uniaxial tensile tests or high-resolution ultrasound techniques^14–18^. Several studies showed that keratoconus cornea stands in contrast to the healthy cornea, both in regard to mechanics and collagen structure^19,20^. Specifically, it has been proposed that collagen structure varies axially in keratoconus cornea compared to the healthy cornea^21,22^. As the collagen structure is directly tied to the biomechanical properties, it is expected that mechanical properties will vary in the axial direction^23^.

Numerical models are intended to contribute to future *in-vivo* biomechanical analysis. There have been a significant number of studies which have developed the constitutive models of the non-linear viscoelastic behavior of soft tissues. Hamilton-Zabolotskaya separated the effects of compressibility and shear deformation by expanding a fourth-order isotropic elastic energy density introduced by Landau and Lifshitz^24,25^. Destrade et al. (2010) demonstrated that the third-order behavior of incompressible solids with parallel fibers can be described by 7 elastic constants^26^. Ye et al. (2018) introduced a finite element formulation to show the non-linear behavior of finite amplitude shear waves in soft solids, considering a visco-hyperelastic Landau’s model^27^.

Some other models were developed to describe the mechanical behavior of cornea in quasi-static and dynamic states. Alastrué et al. (2005) considered a nonlinear anisotropic hyperelastic behavior model of the cornea that strongly depends on the physiological collagen fibril distribution under a finite element context^28^. Pandolfi and Holzapfel (2008) proposed a three-dimensional computational model for the human cornea that was able to provide the refractive power by analyzing the structural mechanical response with the non-linear regime and the effect of the intraocular pressure^29^. Elsheikh et al. (2010) employed non-linear finite element analysis of corneal models to assess the importance of considering the cornea’s hyperelastic, hysteretic and anisotropic behavior, multi-layer construction, weak inter-lamellar adhesion, non-uniform thickness, elliptical topography, and connection to the sclera^30^. Nguyen et al. (2010) developed an inverse finite element method to determine the anisotropic properties of bovine cornea from an in-vitro inflation experiment^31^. Su et al. (2014) derived a corneal hyper-viscoelastic model to describe the material properties more accurately, and explained the mathematical method for determination of the model parameters^32^. Whitford et al. (2017) introduced the combination of the complex anisotropic representation, shear stiffness and regional variation of fibril density of the human cornea with its viscoelastic behavior. The study further attempted to calibrate the proposed model with existing ex-vivo human data^33^.

Despite of the capability to describe part of the mechanical behavior of the cornea, none of these models can fit the non-linear corneal mechanical behavior with R^2^ > 0.9, lacking discriminative parameters for the description of structural changes under non-linear models with P < 0.05. Therefore, there is a critical need to find a model that can highly fit to the non-linear mechanical behavior of the cornea. The objective of this study was to evaluate which non-linear model could best describe the mechanical behavior of the corneal tissues, including the characterization of the discriminative capability of the model to distinguish the structural changes between the healthy and damaged corneas. To this purpose, three hyperelastic models (i.e. Hamilton-Zabolotskaya, Ogden and Mooney-Rivlin models) were fitted to the stress–strain results from healthy and NaOH-treated corneas in order to obtain the non-linear model parameters.

## Materials and methods

### Sample preparation

**Figure 1:**
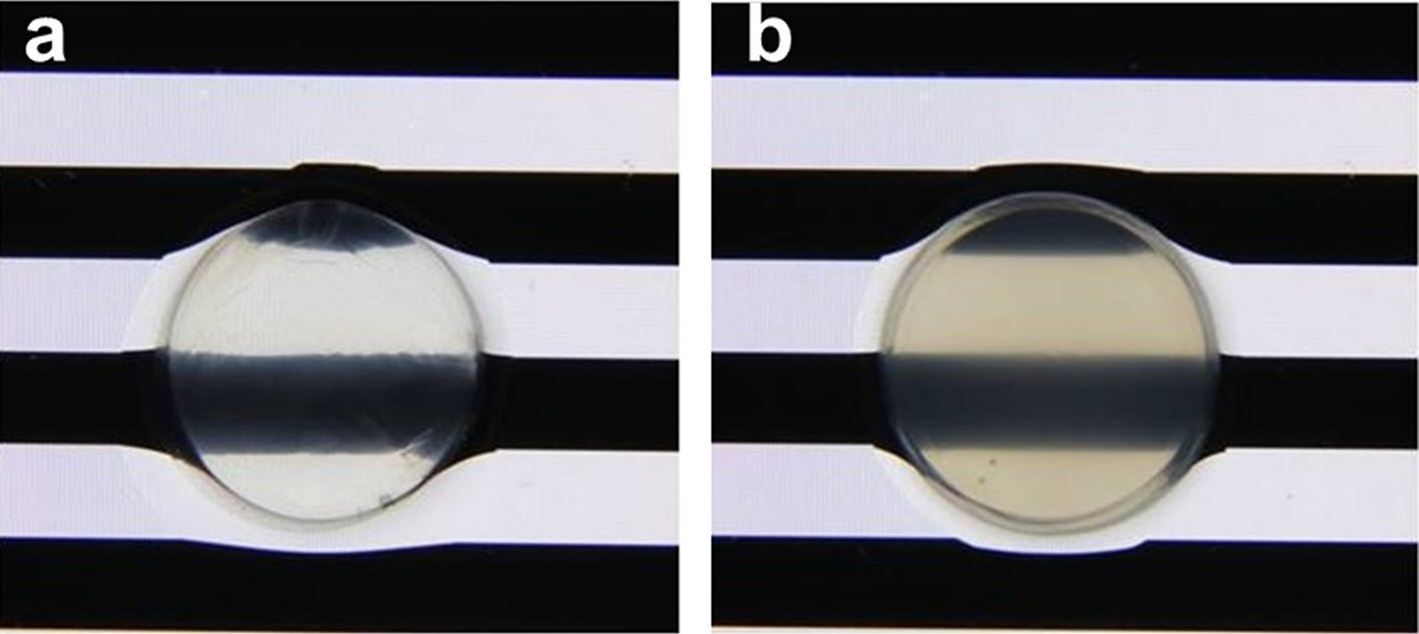
Representative pictures of the non-treated (**a**) and NaOH-treated (**b**) corneas, after obtaining the central corneal button by trephination. Corneal buttons were placed on a standardized black and white band illuminated pattern in order to show the changes on transparency after the alkali burn.

Due to the limitations of human corneal tissue sampling, porcine models have been widely used for understanding physiological changes of the healthy and damaged tissue. The similarity of structural components of porcine samples to the human ones, results in a good insight to the mechanical behavior of the cornea^34^. The porcine eye globes were taken immediately post-mortem from a local slaughter house. The eyes were kept in a lab freezer at − 20 °C to avoid tissue degradation until being prepared for the tests. The samples were excised from the eye globe with 2 mm of sclera. Two different groups were prepared (n = 8): control (non-treated) and NaOH-treated (damaged) groups (Fig. 1). For the NaOH-treated group, corneal buttons were soaked in 1.5 M NaOH for 2 min, followed by washing using water and phosphate buffer saline (PBS) each for 2 min, independently. Alkali solution causes a chemical burn to the corneal tissue, simulating a disease associated to structural damage of the stromal layer^35^. Afterwards, tissue strips were prepared by a custom made bladed punch. All the tissue strips were only excised in temporal-nasal direction in order to avoid anisotropic variations of mechanical properties.

### Uniaxial tensile tests

**Figure 2:**
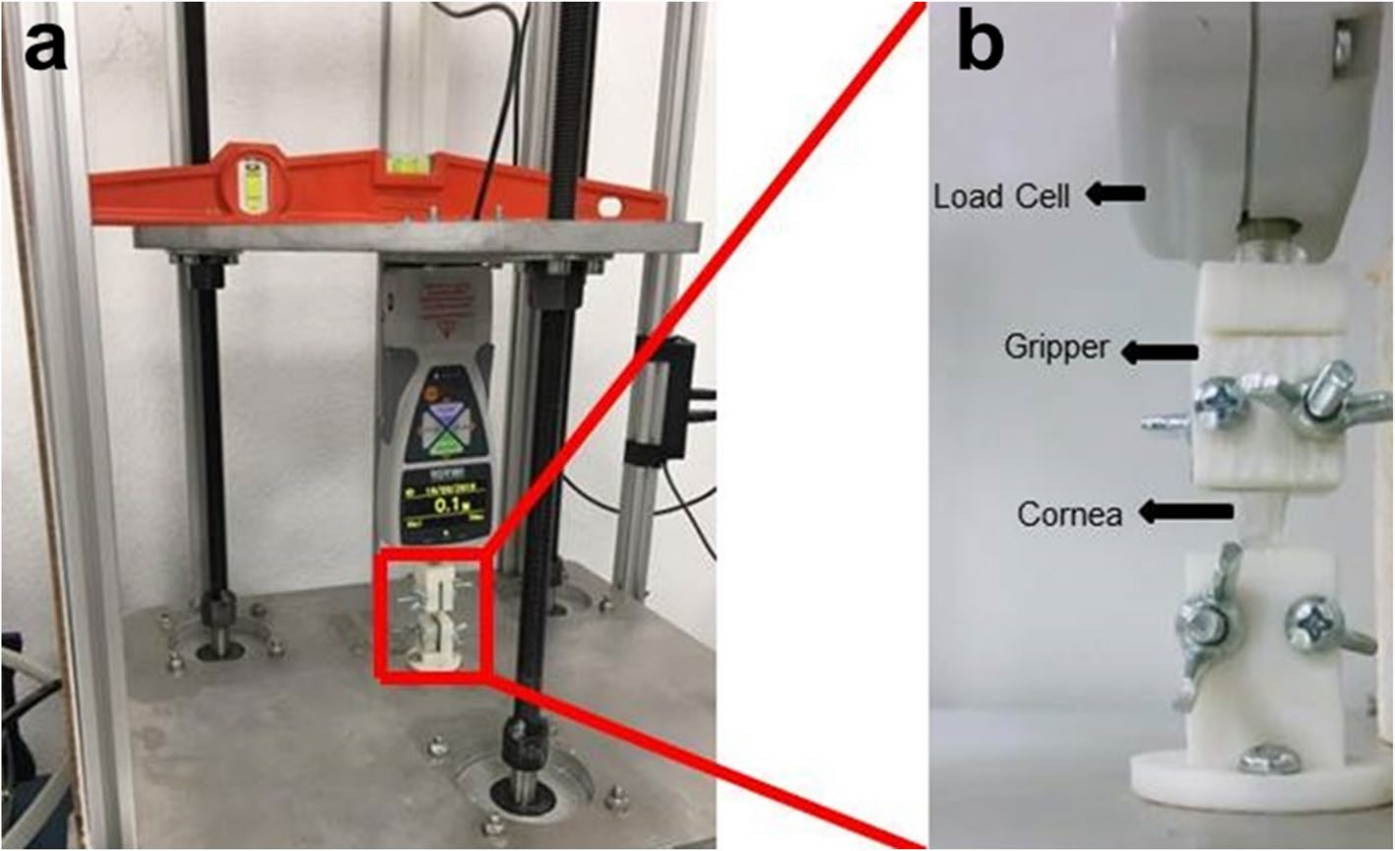
The front view of the uniaxial tensile setup (**a**) and its lateral (camera) view (**b**).

The stress–strain behavior of the two groups of corneal tissues was measured by using a custom made uniaxial tensile machine (Fig. 2a). The uniaxial tensile machine was designed, fabricated and calibrated for the mechanical characterization of soft tissues at the Ultrasonics Lab, University of Granada, Spain. Force was measured using an Imada ZTA-500 force measurement system, connected to a PC by USB and an automated Matlab code to record and analyze the force results with an accuracy of ± 0.2%, and displacement was obtained through the movement of the uniaxial tensile stepper motors at each increment with precision less than 5 microns (Fig. 2b). The displacement was measured by averaging the deformation of the complete probe, while the reality is that the deformation will concentrate on the narrow part of the probe. Therefore, the limitation is that the clamps and the parts of the specimen close to the clamps were neglected, which implies a bias (non-random error), which should not alter the hypothesis testing purposes, as they affect like a proportional constant across all the tests, and therefore is canceled-out at the hypothesis testing. A high resolution camera, IPEVO Ziggi-HD High Definition USB CDVU-04IP model 5MPix 4:3 ratio 2,560 × 1,920, was used to monitor the tissue deformation but not to measure displacements. The uniaxial tensile tests were performed immediately after preparing the corneal strips from the two groups in order to avoid changes in the mechanical response of the tissue.

Prior to the experiments, the length, width and thickness of the corneal strips were measured using a digital caliper (Table 1). No significant differences were found between the groups, while the samples length–width ratio was not enough low to be concerned about clamping issues^36^. The samples were pre-conditioned at a low strain regime, namely 5%, to gain the mechanical response of the corneal tissue close to in-vivo conditions^37^. The tissues underwent a quasi-static uniaxial tensile displacement to the rupture point at a rate of 0.2 mm/s. This testing technique is unable to represent the in-vivo condition. However, it is suitable for finding which hyperelastic model could best describe the non-linear mechanical behavior of the cornea. No significant dehydration of the samples was observed during the tests. The tensile stress and strain were calculated by dividing the measured force, maximally 24.6 N and 15 N for the control and damaged samples respectively, by the initial cross-sectional area and the displacement by the initial length of the strips, respectively^38,39^. The thickness of the samples was considered constant during the test.

**Table 1:** The average measured dimensions of the samples in different groups.

Groups	Ave. length (mm)	Ave. width (mm)	Ave. central thickness (mm)
Control group	16.02 ± 1.33	5.24 ± 1.67	1.56 ± 0.22
NaOH-treated group	13.12 ± 2.45	5.05 ± 0.97	1.76 ± 0.21
P-value	0.16	0.789	0.1

### Mechanical analysis

The tensile stress and strain were calculated using the force and displacement measured in the tests as functions of time. Curve fitting tool in MATLAB R2018a, using the non-linear least squares method, was implemented to fit best non-linear models to the stress–strain curves. The material parameters were adjusted by defining ranges of values, according to the literature, in order not to obtain negative or non-realistic parametric values, while the least squares method is the algorithm that solves the inverse problem of fitting a hyperelastic model to the experimental data by minimizing a residue^40^.

The elastic response of the tissues was described by the three hyperelastic models: Hamilton-Zabolotskaya model, Ogden model and Mooney-Rivlin model (Eqs. 1, 2 and 3, respectively)^24,41,42^.

1 $${\text{E }} = \, \mu \cdot{\text{I}}_{{2}} + \, \left( {{1}/{3}} \right)\cdot{\text{A}}\cdot{\text{I}}_{{3}} + {\text{ D}}\cdot{\text{I}}_{{2}}^{{2}}$$

2 $$f = \sigma \cdot\lambda^{ - 1} = \, \mu \cdot(\left( {\lambda^{\alpha - 1} } \right) \, - \, \left( {\lambda^{ - \, (1 + 0.5\alpha )} } \right)$$

3 $$\sigma \, = { 2}\cdot\left( {\lambda^{{2}} {-}{ 1}/\lambda } \right)\cdot({\text{C}}_{{1}} + {\text{ C}}_{{2}} \cdot{1}/\lambda )$$

where in Eq. (1), E is a strain energy function; coefficient μ is the shear modulus, parameters A and D indicate the third and fourth order elastic constants of the stress–strain curves and I_2_ and I_3_ are the second and third order lagrangian strain invariants.

In Eq. (2), λ indicates the stretch ratio, μ and α are the shear modulus coefficients.

Where in Eq. (3), λ, C_1_ and C_2_ indicate the stretch ratio and the Mooney–Rivlin material constants, respectively.

### Statistical analysis

A coefficient of determination R^2^ was considered for the acceptance of the models fitted to the experimental results. The minimum R^2^ = 0.9 was considered for the acceptance of the hyperelastic models fitted to the two sample groups^38,39^. Two samples T-test, using MATLAB R2018a function ttest2, was implemented to study the significance of the non-linear model parameters, as well as dimensions of samples, for the structural changes between the two sample groups. A value of p < 0.05 was considered statistically significant for the differences of the non-linear parameters between the control and NaOH-treated groups. n.s., *, **, ***, and **** represent p greater than 0.05, p < 0.05, p < 0.01, p < 0.001 and p < 0.0001, respectively. The estimated coefficients were represented graphically with box plots. Additionally, the Pearson correlation coefficients for the model parameters between the two groups were calculated.

## Results

**Figure 3:**
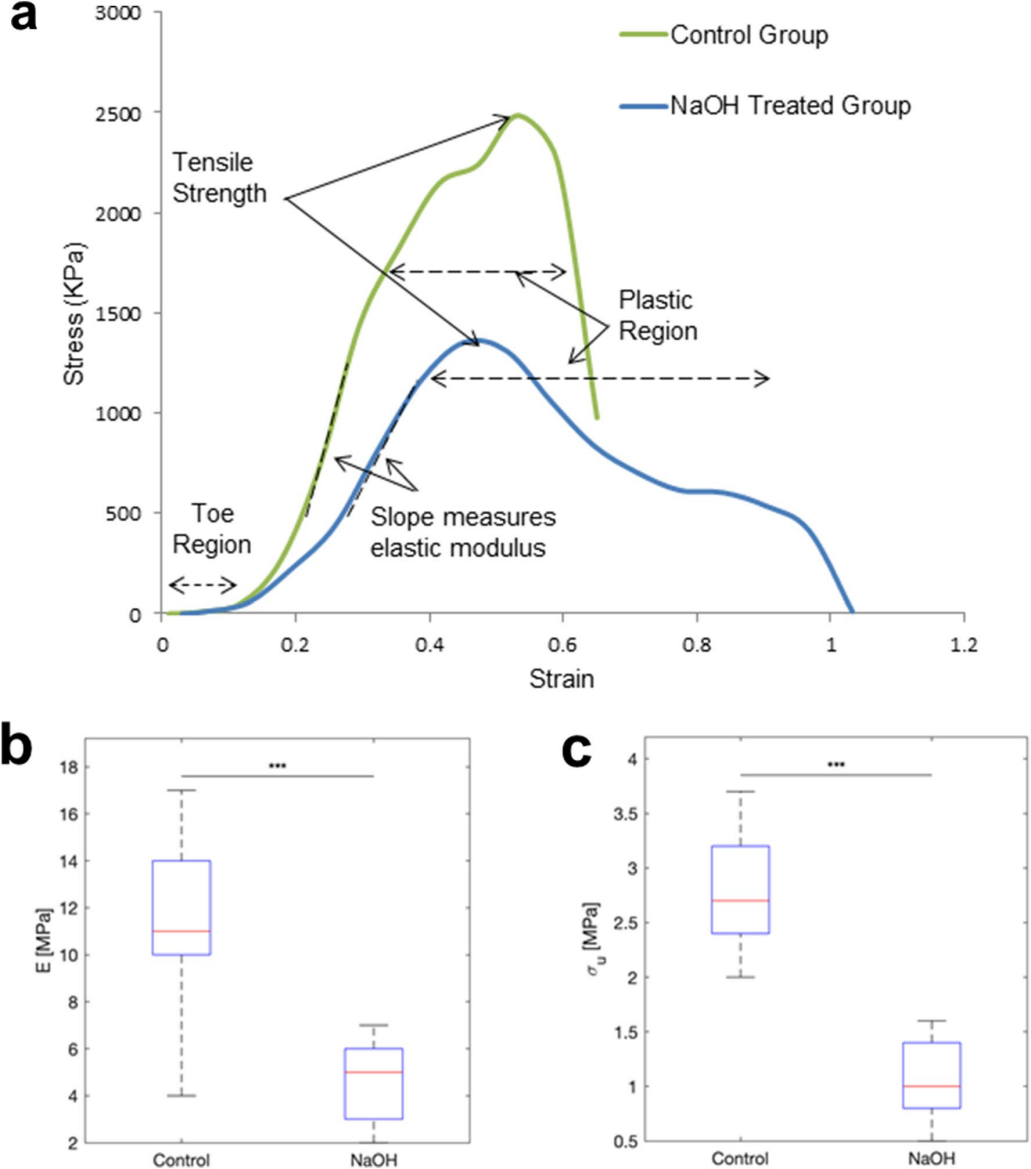
Representative stress–strain curves for the two groups (control versus NaOH-treated group) (**a**). Elastic modulus (**b**) and tensile strength (**c**) were significantly different between the groups (p = 0.0005).

The uniaxial tensile tests were performed to the rupture point on the two groups of 8 corneal samples, control and NaOH-treated, as shown in Fig. 3a. The strain data were acquired immediately before the initial resistance from the tissue was observed. Fitting the non-linear models requires estimating all the parameters including linear modulus, where it is an important parameter to be explored in order to validate the results. A significant difference was observed in the elastic modulus, which was defined as the slope of the tangent to the stress–strain curves at the initial stage of elastic region, between the two groups (p = 0.0005) (Fig. 3b), as well as the tensile strength (p = 0.0005) (Fig. 3c).

**Figure 4:**
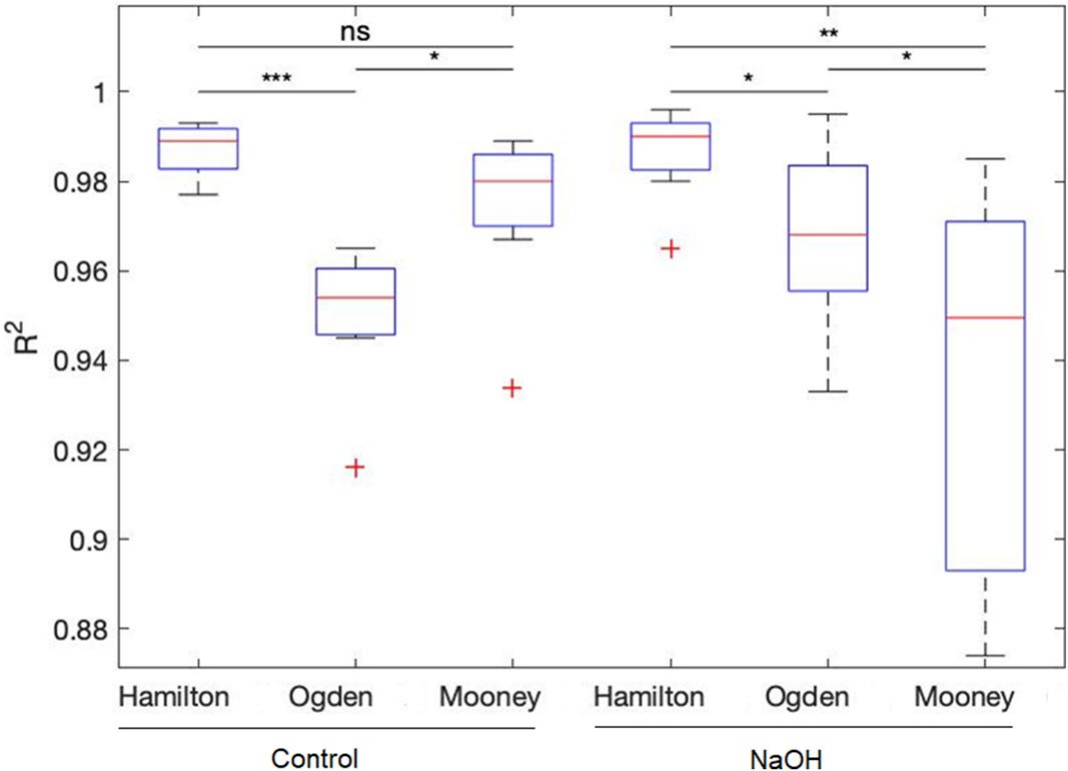
The coefficient of determination R^2^ of the three hyperelastic models fitted to the elastic response of the control and NaOH-treated groups.

The coefficient of determination R^2^ of the three hyperelastic models fitted to the elastic response of the two groups (Fig. 4). A minimum R^2^ of 0.9 was considered for the acceptance of the best models fitted.

**Figure 5:**
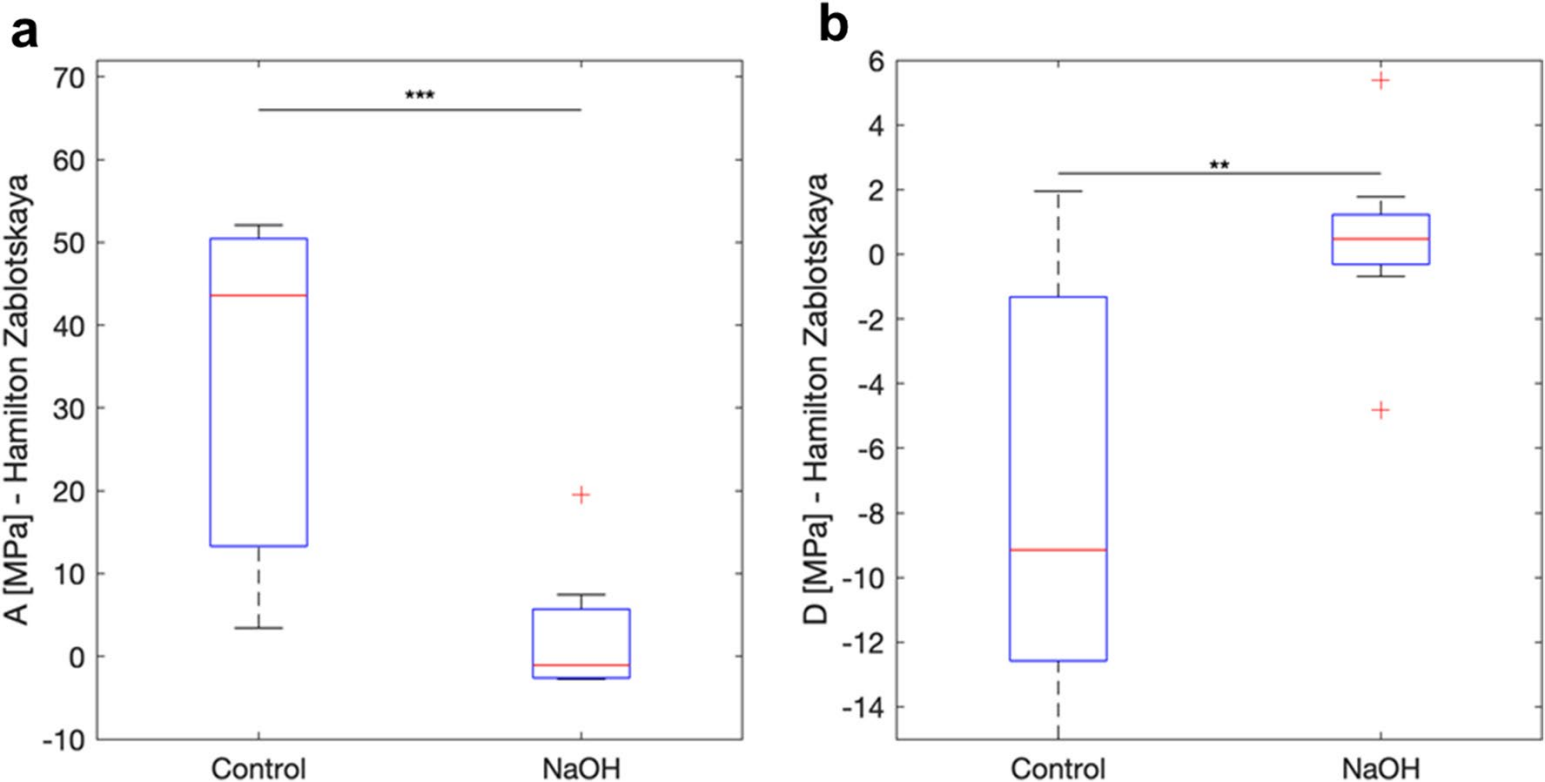
Hamilton–Zabolotskaya model. The parameter A was significantly different between the two groups (p = 0.0005) (**a**). The parameter D difference between the two groups was significant (p = 0.0045) (**b**).

For the Hamilton-Zabolotskaya model, the difference between the third order elastic constant (parameter A) for the two groups was found to be significant (p = 0.0005) (Fig. 5a). The difference between the fourth order elastic constant (parameter D) for the two groups was significant, as well (p = 0.0045) (Fig. 5b).

**Figure 6:**
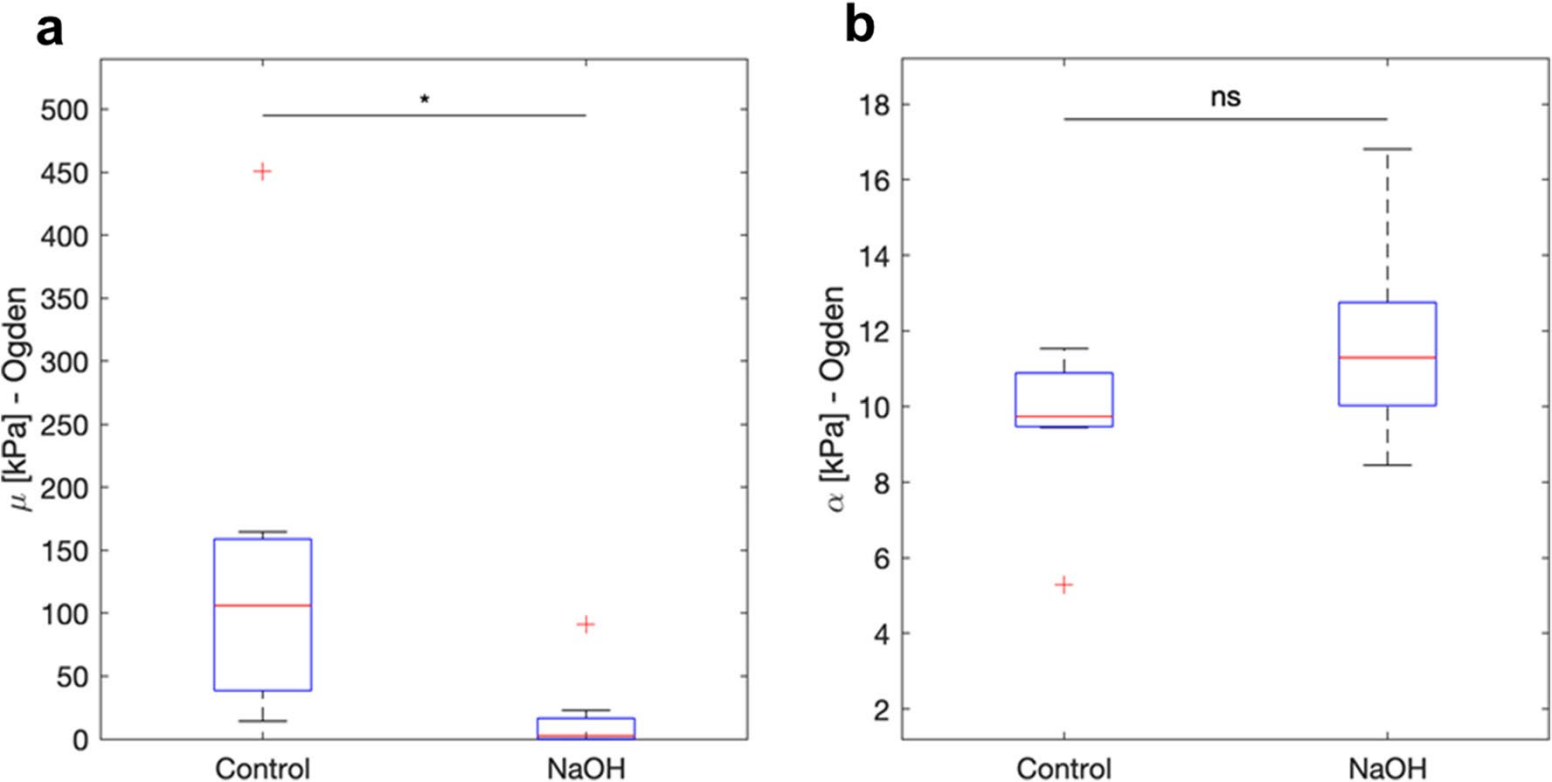
Ogden model. The coefficient Mu (μ) was significantly different for the two groups (p = 0.017) (**a**). The coefficient Alpha (α) was different but not significant for the two groups (p = 0.055) (**b**).

For the Ogden model, the coefficient μ was found to be significantly different for the two groups (p = 0.017) (Fig. 6a). The coefficient α was different between the two groups (p = 0.055) (Fig. 6b).

**Figure 7:**
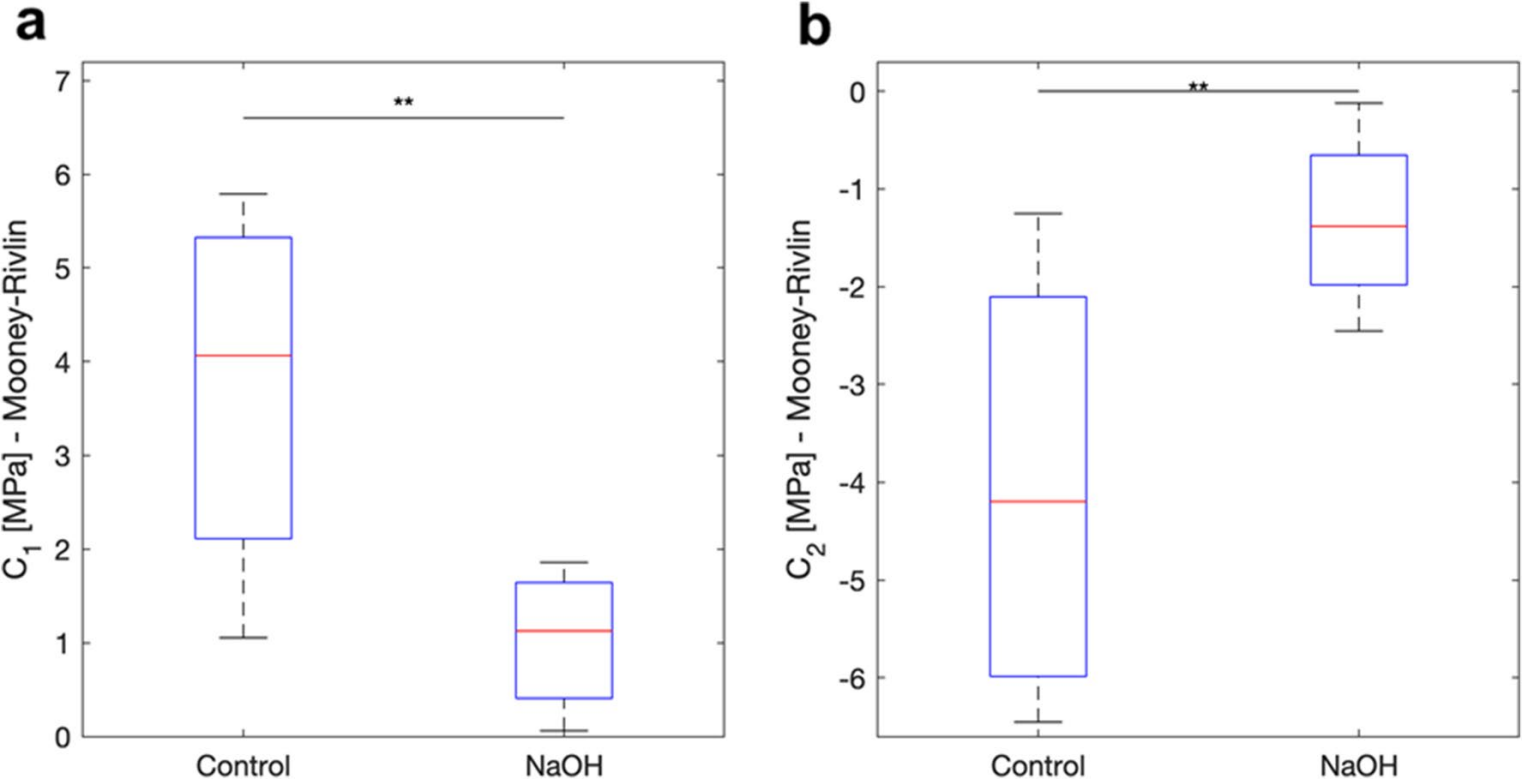
Mooney–Rivlin model. The coefficient C1 was significantly different for the two groups (p = 0.001) (**a**). The coefficient C2 was also significantly different for the two groups (p = 0.002) (**b**).

For the Mooney-Rivlin model, the material constants C_1_ and C_2_ were significantly different for the two groups, (p = 0.001) and (p = 0.002), respectively (Fig. 7).

The mean and standard deviation of the non-linear parameters of the three hyperelastic models for the two groups are shown in Table 2.

**Table 2:** Non-linear parameters of the three hyperelastic models (mean ± standard deviation).

Non-linear parameters	Parameter A	Parameter D	Coefficient μ	Coefficient α	Constant C_1_	Constant C_2_
Control group	34,153.42 ± 20,918.87	− 7,796.18 ± 6,974.51	142.36 ± 147.47	9.58 ± 2.04	3,706 ± 1816.18	− 4,122.42 ± 2,102.4
NaOH-treated group	1,054.52 ± 9,926.87	416.87 ± 2,811.81	16.24 ± 31.27	11.67 ± 2.59	1,034.24 ± 686.98	− 1,327.61 ± 287.44
P-value	0.0005	0.0045	0.017	0.055	0.001	0.002

The Pearson correlation coefficients between the two groups were calculated for each parameter of the three hyperelastic models (Table 3).

**Table 3:** Pearson correlation coefficients between the two groups.

Pearson correlation coefficients (r)	Parameter A	Parameter D	Coefficient μ	Coefficient α	Constant C_1_	Constant C_2_
Control group–NaOH-treated group	− 0.013	− 0.344	− 0.105	− 0.578	0.163	− 0.020

In addition, the Pearson correlation coefficients between the model parameters of each hyperelastic model were calculated (Table 4).

**Table 4:** Pearson correlation coefficients between the model parameters of each hyperelastic model.

Pearson correlation coefficients (r)	Parameters A and D	Coefficients μ and α	Constants C_1_ and C_2_
Control group	− 0.932	− 0.962	− 0.987
NaOH-treated group	− 0.967	− 0.613	− 0.976

## Discussion

Quantifying the non-linear mechanical parameters of soft tissues has the potential to become specific diagnostic criteria for some corneal diseases, due to the fact that non-linear mechanical characterization and non-linear model parameters are very sensitive realistic approaches to measure the tissue structural damages, i.e. expressing larger variations than linear ones. In tensile testing, the non-linear behavior is apparent at high strains, but there are emerging elastography and probing technologies that precisely quantify non-linearity at extremely low strains. For instance, harmonic generation technique measures the amplitude of the harmonics when tissue is excited with ultrasound at strains of the order of 10^−5, and these harmonic amplitudes are proportional to the non-linearity^43–45^. There have been a significant number of studies which have improved the non-linear models of corneal tissue biomechanics. Nevertheless, this was the first work to describe the non-linear mechanical behavior of the corneal tissue including the characterization of the discriminative capability of the model to distinguish structural changes between the healthy and damaged cornea.

Hyperelastic models are suitable for characterization of non-linear mechanical behavior of soft tissues involving large deformations. Hyperelastic constitutive laws are used to model materials that respond elastically when subjected to very large strains. They account both for nonlinear material behavior and large shape changes^46^. The non-linear elastic response of the corneal tissue was well described by the three hyperelastic models: Hamilton–Zabolotskaya model, Ogden model and Mooney–Rivlin model. The Hamilton–Zabolotskaya model was best fitted to the stress–strain results for the two sample groups with the highest coefficient of determination R^2^. It was proved that the third and fourth order elastic constants could best characterize the non-linear mechanical behavior of the control and damaged corneal tissues.

The Hamilton–Zabolotskaya model constants were measured and compared for the two sample groups. A significant difference in the third and fourth order elastic constants (parameters A and D) between the two sample groups, p = 0.0005 and p = 0.0045, respectively, was in accordance with previous results related to corneal stromal damage^14,23^. This resulted in a significant decrease in the tensile strength of the tissue. A negative correlation coefficient was found for parameters A and D between the two sample groups, indicating the differences in the structural behavior of control corneas versus NaOH-treated ones. This suggests that the damage caused to the stromal layer after treating with NaOH led to alterations in the collagen architecture, showing similar mechanical effects to those observed in keratoconus^14,23^.

The Ogden model showed a significant difference in the coefficient μ between the two sample groups, (p = 0.017), which could be related to different facts. Specifically, the shear strength was considerably decreased for the NaOH-treated samples possibly due to the disorganization of the tissue structure^14,23^. The coefficient α increased for the NaOH-treated samples (p = 0.055), indicating a loss in the shear stiffness of the tissues. This loss could be related to the disruption of lamella interweaving and collagen crosslinking^19,23^. A negative correlation coefficient was observed for coefficients μ and α between the two sample groups, indicating the structural changes as well as the differences in structural behavior of the two groups.

The Mooney–Rivlin material constants C_1_ and C_2_ were obtained for the control and the NaOH-treated groups. The material constant C_1_ was considerably different between the two groups (p = 0.001). It was considerably decreased for the NaOH-treated samples possibly due to the structural damages within the tissue caused by the alkali solution. The material constant C_2_ was significantly different for the two groups (p = 0.002), while increased for the NaOH-treated samples compared to the control ones. This increase indicates a loss in the stiffness of the tissues which could be related to the structural disorganization in the collagen fibrils^18–20,23^. A negative correlation coefficient was found for constant C_2_ between the two sample groups.

The measurements of the parameters (A and D) involved in Hamilton–Zabolotskaya model represent the best fit explaining the statistical differences between the two groups (p = 0.0005 and p = 0.0045), respectively. The statistical significance of Mooney–Rivlin parameters (C_1_ and C_2_) is quantified determining the difference between the analyzed groups with p-values of p = 0.001 and p = 0.002, respectively, after applying an intermediate adjustment. Finally, the Ogden model has a low significant value in this study for the model parameters (Mu and Alpha) explaining the statistical differences between the two groups (p = 0.017 and p = 0.055), respectively.

In conclusion, quantification of non-linear model parameters of control and damaged corneal tissue is correlated to the changes in the tissue structure and its effect on the mechanical behavior of the cornea.
